# Uses of Bibliometric Techniques in Public Health Research

**Published:** 2017-10

**Authors:** Luana Kelle Batista MOURA, Rafael Fernandes de MESQUITA, Mitra MOBIN, Francisca Tereza Coelho MATOS, Thiago Lima MONTE, Eliana Campelo LAGO, Carlos Alberto Monteiro FALCÃO, Maria Ângela de Arêa Leão FERRAZ, Tanit Clementino SANTOS, Laelson Rochelle Milanês SOUSA

**Affiliations:** 1. Dept. of Dentistry, School of Dentistry, Center UNINOVAFAPI, Teresina, Brazil; 2. Dept. of Administration, School of Administration, Federal Institute of Piauí, Teresina, Brazil; 3. Dept. of Administration, School of Administration Potiguar University, Natal, Brazil; 4. Dept. of Nursing, School of Nursing, Federal University of Piauí, Teresina, Brazil

## Dear Editor-in-Chief

Bibliometric research uses statistical and mathematical methods to analyze and build indicators on the dynamics and evolution of scientific and technological information on a given topic.

The relevance of its applicability as a data collection and analysis technique has been corroborated as one of the argumentative sources in the research for investment resources in academic rankings (1–2).

To carry out a bibliometric study, it is important to consider the selection of the database to be used; maintaining compatibility of this choice with the research aims and reach the results (3). In this way, the ISI, Web of Knowledge, Web of Science database is recommended for its academic recognition of being considered one of the most comprehensive databases of journals covering several areas of scientific knowledge (4), besides being important and pioneering in bringing together journals of more than one hundred areas of knowledge.

Data collection from the ISI Web of Knowledge/Web of Science^TM^ database can be done by exporting this data to the HistCite^TM^ bibliometric analysis software package to organize information and facilitate analysis.

The data can be analyzed considering: the annual evolution trajectory of the publications; the journals with the greatest number of records; the authors with more publications; the number of papers distributed by author’s country of origin; the most cited articles in the Web of Science (global) and those most cited in the set of selected articles (local).

Thus, investigations of academic and practical problems of public health research can be developed in a methodological approach that maps the scientific production of several fields and provides researchers with sources with recognized value through citation and authoring metrics: bibliometric techniques (5, 6). Although bibliometric rely on techniques that go beyond citation analysis, bibliometric are currently based on the understanding of the production of knowledge in a scientific field or on specific themes and the activity of its producers (7).

The understanding of knowledge production and its measurement are supported and accompanied by the evolution of digital communication technologies and this allows a greater reach of information (8).

The suggestion that we address in this letter is the proposal of answers to the research problems related to the most varied subjects of public health through research in international databases that seek to map the international scientific production. This map ensures the understanding of the knowledge produced in a given field and allows monitoring its development, implying improvements in the development of public health policies.
